# A health communication intervention to integrate partner testing with antiretroviral therapy service among men who have sex with men in China: an observational cohort study

**DOI:** 10.1186/s12889-018-6147-5

**Published:** 2018-11-06

**Authors:** Yuting Lian, Yan Zhao, Joyce Wang, Cynthia Shi, Kerong Wang, Xia Wang, Guohui Wu, Hongxia Wei, Xiaoli Wei, Yan Luo, Min Zhao, Zunyou Wu

**Affiliations:** 1 National Center for AIDS/STD Control and Prevention, Chinese Center for Disease Control and Prevention, 155 Changbai road, Changing district, Beijing, 102206 China; 2 Harvard Medical School, 25 Shattuck Street, Boston, MA 02115 USA; 3 Center for Interdisciplinary Research on AIDS and Department of Epidemiology of Microbial Diseases, Yale School of Public Health, 135 College Street, New Haven, CT USA; 4 Beijing Ditan Hospital, Capital Medical University, Beijing, China; 5 Wuhan Center for Disease Control and Prevention, Wuhan, China; 6 Chongqing Center for Disease Control and Prevention, Chongqing, China; 7 Nanjing Center for Disease Control and Prevention, Nanjing, China; 8 Xi’an Center for Disease Control and Prevention, Xi’an, China; 9 Hangzhou Center for Disease Control and Prevention, Hangzhou, China; 10 302 Military Hospital of China, Beijing, China

**Keywords:** HIV testing, Partner testing, Men who have sex with men, Case manager

## Abstract

**Background:**

In China, antiretroviral therapy (ART) clinics focus on treating people living with HIV and are not required to undertake testing of high-risk populations. To improve partner testing among MSM, we implemented a health communication pilot intervention integrating partner testing with ART services. We aimed to assess the feasibility of the partner referral service and identify the predictors of both successful partner referral for HIV testing and HIV-positive test results among referred partners.

**Methods:**

This program ran from April 2014 through December 2015 at designated ART clinics in six cities. The index participants, men living with HIV enrolled at an ART clinic, were assigned a case manager who assumed responsibility for routine ART-related counseling and mobilization of HIV-positive index participants for partner referral testing. Case managers were either nurses or contract staff. The successful referral rate was the proportion of index participants who referred a sexual partner for HIV testing. The HIV-positive partner rate was the proportion of the newly referred contacts who tested HIV-positive. Factors associated with the successful referral rate and the HIV-positive partner rate were assessed.

**Results:**

Two thousand three hundred eighty-two index participants were enrolled. The median age was 30 years (IQR 26–37). 829index participants (34.80%) successfully referred at least one sexual partner for screening, and 92 (11.10%) referred partners were HIV-positive. Having a hospital nurse as case manager was associated with both successful partner referral (AHR = 1.56, 95% CI = 1.36–1.80) and having a HIV-positive partner (AHR = 2.35, 95% CI = 1.45–3.92). Index participants who were married (AHR = 1.44, 95% CI = 1.20–1.73) or employed (AHR = 1.29, 95% CI = 1.11–1.49) were more likely to successfully refer a partner for testing. Stable male partner relations were more likely to result in a referred partner testing HIV-positive (AHR = 5.50, 95% CI = 1.85–16.39).

**Conclusion:**

Our findings indicated that integration of MSM partner testing with ART services via health communication was feasible. Nurses as case managers effectively encouraged index participants to refer their sexual partners for HIV testing.

## Background

Over the last 10 years, the incidence of adult HIV infection has declined by at least 50% in 146 countries around the world [1]. But key populations—including sex workers, intravenous drug users, transgender people, and men who have sex with men (MSM)—remain at much higher risk of HIV infection [1]. Studies suggest that MSM are 24 times more likely to acquire HIV than other adult males. In order to curb this epidemic, UNAIDS established the “90–90-90” targets in 2014 [2, 3]. This strategy calls for 90% of people living with HIV to be diagnosed, 90% of diagnosed patients to be sustained on antiretroviral therapy (ART), and 90% of treated patients to achieve viral suppression by 2020. As the entry point of the care cascade and the prelude to the “treat all” strategy, the first 90% is the cornerstone of the 90–90-90 approach. Approximately 40% of people living with HIV around the world are still unaware of their serostatus [1].

The HIV epidemic in China has a low national prevalence at 0.06% (0.05–0.07%) of the total population [4]. Currently, sexual transmission is the primary mode of HIV transmission, and specific regions and populations bear a heavier burden. In particular, MSM are disproportionally affected by HIV and have experienced a sharp rise in incidence in recent years. Among newly diagnosed HIV cases nationwide, the percentage of all sexually transmitted cases increased from 33.10% in 2006 to 92.20% in 2014, and the proportion of HIV cases among MSM increased from 2.50% in 2006 to 25.80% in 2014 [5]. The inconvenience of HIV testing, social stigma, and lack of knowledge about HIV are barriers to HIV testing for MSM [6]. Because same-sex relationships have traditionally been unacceptable in China, HIV-positive MSM have protected their identities more carefully [7, 8]. Therefore, there are certain obstacles to partner testing among MSM due to concerns about privacy and stigma. To deal with the challenge of identifying more cases among MSM, a variety of prevention and treatment interventions have previously been implemented, most notably testing by community mobilization [9].

Partner referral testing has been a powerful tool for identifying cases among heterosexual couples in China, with 87.70% of married people living with HIV referring their spouse for testing [10]. Accordingly, in this study, partner testing services were applied to MSM as a potential intervention [11, 12]. Previously, when a similar strategy was implemented in two Chinese cities (Kunming and Hangzhou) via community mobilization, about 21.80% of MSM sexual partners received HIV testing and10.90% tested HIV-positive [13].

In China, ART clinics only focus on treating people living with HIV and are not required to actively undertaketesting of high-risk populations. HIV testing has typically been provided in voluntary and confidential counseling and testing clinics, different levels of health organizations, and community-based organizations. When HIV infection was identified, individuals were referred from these testing facilities to ART clinics at designated provider hospitals [14, 15].

In order to address the challenge to HIV case finding posed by this sectorial division of labor, we conducted a novel health communication-based intervention to integrate MSM partner testing with ART clinic services. For each HIV infected index participant who was newly referred to an ART clinic, we assigned a case manager who oversaw treatment and care, with emphasis on additional partner testing services. This pilot study aims to assess the feasibility and acceptability of the integrated partner testing service for improving case finding among MSM. We identified the predictors of both successful partner referral for HIV testing and HIV-positive test results among referred partners.

## Methods

### Study design and intervention

The study was launched by the National Center for AIDS/STD Control and Prevention (NCAIDS) of the national Chinese Center for Disease Control (China CDC) in April 2014. ART clinics were enrolled from six cities: Beijing, Nanjing, Xi’an, Chongqing, Hangzhou, and Wuhan. These cities were selected to ensure variation in geography and characteristics of the local epidemics. The endpoint of observation was December 31, 2015.

Under Chinese existing standard-of-care procedures, ART clinics were only responsible for providing ART to HIV-positive individuals. In this pilot program, partner testing services were integrated into the ART clinics by assigning a trained case manager to each enrolled HIV-positive index participant. Case managers assumed responsibility for mobilizing HIV-positive participants to refer partners for testing, with the goal of improving case-finding ability.

Based on the personnel availability of the ART clinics, we selected nurses as case managers in three sites, and other case managers were recruited as contract staff if clinics could not appoint nurses for involvement. The same funding was provided to all clinics. Of a total 10 case managers, six were nurses who were already employed at the clinic and four were contract staff who were newly hired for this pilot. Selection of contract staff was performed by posting a position notice in project clinics. Candidates qualified in current clinic volunteers and were subsequently approved by the project leader after interview. The educational backgrounds of the contracted case managers varied from sociology to health management, and they all had HIV-related volunteer experience. All case managers were engaged full-time in this program.

This intervention aimed to leverage the potential of good health communication. Neither nurses nor contract staff had previous experience with partner testing mobilization. For all case managers, a two-day training was held at the beginning of the program with follow-up trainings every 3 months. Study investigators and HIV care experts conducted one-on-one training sessions with each case manager on case counseling, HIV treatment and care, partner notification, and especially partner referral testing. Training sessions included simulated case manager-participant interactions. Training materials and operation study manuals were provided by NCAIDS. After the first training and before program initiation, all case managers passed project tests to ensure that they had the ability to execute project procedures. A handbook was issued to all providers on HIV-related counseling and project procedures.

Counseling for mobilizing partner testing was required to be provided for each index participants at the same day of ART initiation and was repeated at 0.5, 1, 2 and 3 months after ART initiation, once every 3 months thereafter. Each counseling session for mobilizing partner testing lasted at least 30 min. To monitor the intervention quality, site supervisors were stationed in each study site for the first week of the intervention. Subsequently, site visits were continued monthly to monitor fidelity to the intervention protocol.

### Inclusion criteria

This was an observational study of a structural intervention program. Index participants were asked to refer their sexual partners to their case manager, who would coordinate with the referred individual for testing. Index participants were enrolled in the study if they were: 1) a male infected with HIV, 2) self-reported sexual contact with another male within the previous 12 months, 3) newly enrolled at an ART clinic between April 10, 2014 and December 31, 2015, and 4) provided informed consent for study participation. Case managers counseled all index participants to refer their partners with potential HIV exposure for HIV screening.

Partners referred to the case manager (hereafter, “referred partners”) were screened for HIV using an ELISA test. Referred partners with a positive HIV screening result were assumed to be HIV-positive for the purposes of this study (i.e. prior to a confirmatory Western blot test).

### Measured variable and outcomes

Study participants were assigned study identification codes, and all study data were de-identified before analysis. The study was approved by the Institutional Review Board of NCAIDS, China CDC (Approval X150129351).

Information was collected on index participants with respect to their age, education level, occupation, marital status, and initial CD4 count. Case manager type was included as a variable to discern the influence of nurses versus contract staff. The successful referral rate for HIV testing was the proportion of index participants who were able to refer a sexual partner to their case manager for HIV testing. The HIV-positive partner rate was the proportion of the newly-referred contacts who tested HIV-positive. Stable male sexual partners were defined as sexual relationships lasting more than 3 months, and casual male sexual partners meant occasional or sexual relationships lasting fewer than 3 months.

### Statistical analysis

The Cox proportional hazards model was used to estimate the associations between individual and structural variables and the outcome of successful partner referral for HIV testing. Logistic regression was used to assess determinants of positive HIV screening among referred partners.

We obtained adjusted hazard ratios (AHRs), adjusted odds ratios (AORs), and 95% confidence intervals (CIs). All *p*-values presented are two-sided and *P* < 0.05 was indicative of statistical significance. Analyses were performed with SAS 9.2 software (SAS Institute Inc., USA).

## Results

### Characteristics of participants

A total of 2382 HIV-positive index participants were enrolled in this partner testing pilot program: 752 in Beijing, 424 in Nanjing, 422 in Wuhan, 394 in Xian, 212 in Hangzhou, and 178 in Chongqing. The median observational period of all index participants was 0.69 person-years. The median age was 30 years (IQR 26–37). In terms of education, 2047 (85.94%) subjects had completed at least high school. A majority of the participants were single (81.86%), and 1071 (44.96%) were unemployed at the time of enrollment. Most index participants (1337, 56.13%) had an initial CD4 count less than or equal to 350 cells/mm^3^ (Table 1).

**Table 1:** The characteristics of index participants stratified by their case managers type

	Total		Case managers type			
			Nurses		Contract staff	
	*N* = 2382	%	*N* = 1187	%	*N* = 1195	%
Age						
≤ 30	1250	52.48	603	50.80	647	54.14
> 30	1132	47.52	584	49.20	548	45.86
Education						
Junior high school or below	335	14.06	209	17.61	126	10.54
Senior high school or above	2047	85.94	978	82.39	1069	89.46
Marriage						
Single, divorced, or widowed	1950	81.86	910	76.66	1040	87.03
Married	432	18.14	277	23.34	155	12.97
Occupation						
Unemployed	1071	44.96	506	42.63	565	47.28
Employed	1049	44.04	546	46.00	503	42.09
Student	262	11.00	135	11.37	127	10.63
CD4 counts (cells/ul)						
≤ 350	1337	56.13	634	53.41	703	58.83
> 350	1045	43.87	553	46.58	492	41.17

**Fig. 1:**
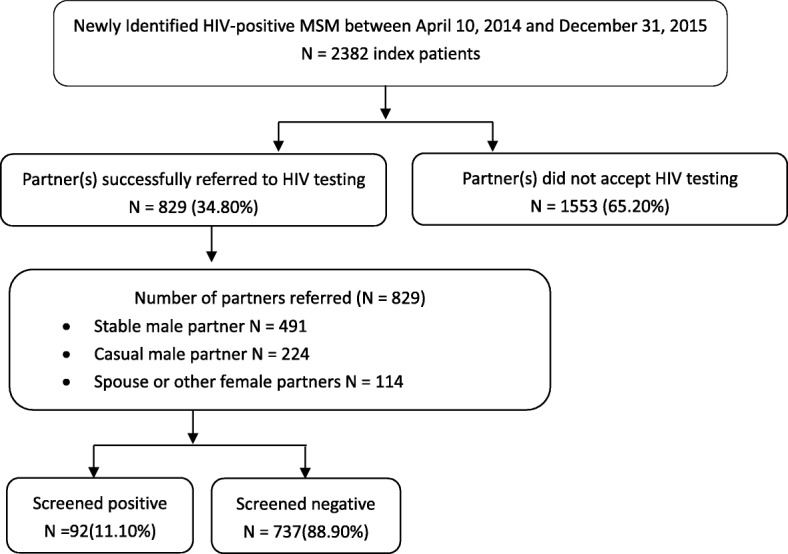
Schematic diagram of study design and partner referral testing procedures

Among all index participants, 829 (34.80%) successfully referred a sexual partner for HIV testing; 106 index participants successfully referred multiple partners for testing, though we selected only the first partner referred for inclusion in our analysis. Among referred partners who were tested, more than half were stable male sexual partners (491/829, 59.23%), followed by casual male sexual partners (224/829, 27.02%) and spouse or other female sexual partners (114/829, 13.75%) (Fig. 1).

### Successful partner referral testing

Cox proportional hazards regression was used to identify factors related to successful partner referral for HIV testing (Table 2). Multivariable analysis further indicated that married index participants were more likely to successfully refer their partners for HIV testing (AHR = 1.44, 95% CI = 1.20–1.73) compared to single index participants. Relative to unemployed index participants, those employed were more likely to successfully refer a sexual partner for testing (AHR = 1.29, 95%CI = 1.11–1.49). Having a case manager who was a nurse increased the likelihood of successful referrals (AHR = 1.56, 95% CI = 1.36–1.80) compared to contract staff.

**Table 2:** Factors associated with successful testing referrals of partners

	Total	Successful Testing	Univariable			Multivariable		
	*N* = 2382	*N* = 829	HR	95% CI	*P* value	AHR	95% CI	*P* value
Age								
≤ 30	1250	412	–	–	–	–	–	–
> 30	1132	417	0.99	(0.83,1.19)	0.937	0.99	(0.84,1.16)	0.906
Education								
Junior high school or below	335	123	–	–	–	–	–	–
Senior high school or above	2047	706	1.13	(0.90,1.42)	0.294	1.13	(0.92,1.38)	0.237
Marriage								
Single, divorced, or widowed	1950	632	–	–	–	–	–	–
Married	432	197	1.46	(1.21,1.77)	<.001	1.44	(1.20,1.73)	, <.001
Occupation								
Unemployed	1071	332	–	–	–	–	–	–
Employed	1049	412	1.29	(1.11,1.52)	0.001	1.29	(1.112,1.49)	0.001
Student	262	85	1.09	(0.83,1.43)	0.558	1.09	(0.85,1.40)	0.495
Case manager type								
Nurse	1187	503	1.37	(1.17,1.61)	<.001	1.56	(1.36,1.80)	<.001
Contract staff	1195	326	–	–	–	–	–	–
CD4 counts (cells/ul)								
≤ 350	1337	448	–	–	–	–	–	–
> 350	1045	381	1.08	(0.93,1.26)	0.326	1.09	(0.95,1.25)	0.211

### Likelihood of referred partners testing HIV positive

Of the 829 referred partners who received testing, 92 (11.10%) screened HIV-positive (Table 3). Stable male partner relations were more likely to result in a referred partner testing HIV-positive (AHR = 5.50, 95% CI = 1.85–16.39). Having a case manager who was a nurse was associated with increased likelihood of referred partners testing HIV-positive, compared to contract staff case managers (AHR = 2.35, 95% CI = 1.45–3.92).

**Table 3:** Factors associated with HIV-positive screens among referred partners

	Testing	HIV-positive		Univariable			Multivariable		
	*N* = 829	*N* = 92	%	OR	95%CI	*P* value	AOR	95%CI	*P* value
Age									
≤ 30	412	46	10.19.	–	–	–	–	–	–
> 30	417	46	11.03	0.99	(0.64,1.52)	0.951	1.01	(0.61,1.69)	0.964
Education									
Junior high school or below	123	17	13.82	–	–	–	–	–	–
Senior high school or above	706	75	10.62	0.74	(0.42,1.30)	0.299	0.80	(0.43,1.49)	0.476
Marriage									
Single, divorced, or widowed	632	73	11.55	–	–	–	–	–	–
Married	197	19	9.64	0.82	(0.48,1.39)	0.458	0.93	(0.48,1.81)	0.834
Occupation									
Unemployed	332	29	8.73	0.58	(0.37,0.94)	0.857	0.66	(0.41,1.08)	0.682
Employed	412	58	14.08	–	–	–	–	–	–
Student	85	5	5.88	0.38	(0.15,0.93)	0.145	0.34	(0.13,0.91)	0.078
Relationship									
Stable male partner	491	69	14.05	4.50	(1.61,13.59)	<.001	5.50	(1.85,16.39)	<.001
Casual male partner	224	19	8.48	2.55	(0.85,7.68)	0.605	2.94	(0.91,9.43)	0.546
Spouse or other female partners	114	4	3.51	–	–	–	–	–	–
Case manager type									
Nurse	503	69	13.72	2.09	(1.28,3.43)	0.003	2.35	(1.45,3.92)	0.001
Contract staff	326	23	7.06	–	–	–	–	–	–
CD4 counts (cells/ul)									
≤ 350	448	50	11.16	–	–	–	–	–	–
> 350	381	42	11.02	0.99	(0.64,1.52)	0.950	0.99	(0.63,1.56)	0.969

## Discussion

The aim of this health communication pilot study was to assess the impact of partner referral testing integration with ART services among MSM in China. We sought to improve case-finding among MSM by training case managers to provide intensified counseling related to partner testing. We found that having a nurse as a case manager, being employed, and being married were more likely to result in successful partner referral testing and HIV-positive screenings among referred partners. Having a stable male partner was also a related factor for referred partners’ screening HIV-positive.

Approximately 35% of index participants referred a sexual partner for testing. This partner referral rate is comparable to other studies. For example, one study reported a 21.8% referral rate in MSM through community-based organizations [13]. Overall, there is still room to improve the HIV testing rate among partners of MSM.

From our study results, we identified a few factors that may highlight underlying social determinants and influence future policy development. Firstly, case managers who were nurses proved to be more effective in mobilizing partner testing [16]. Of index participants who had nurse case managers, 42.38% (503/1187) referred partners for testing, of whom 13.72% (69/503) tested HIV-positive, compared to the 27.28%(326/1195) referral rate and 7.05% (23/326) HIV-positive in the contract staff case manager group. Nurse-delivered interventions had improved outcomes such as ART adherence in another study as well [17]. Despite nurses and contract staff receiving the exact same case manager training, it is possible that nurses were better able to communicate with participants due to their previous health delivery background. Secondly, we also found an association between employment status and the successful referral of a sexual partner, possibly indicating that stable income improved referral for partner testing. Finally, index participants in stable male relationships were more likely to have an HIV-positive partner. This suggests that the partner testing intervention should be scaled up in this population.

Our findings supported sectorial integration as a method to improve case finding, which has proven to be as effective as previous MSM partner testing programs in China [13, 18]. ART clinics already provide patients with regular follow-up and an established continuity of care. Previous integration programs (e.g. tuberculosis and methadone maintenance therapy programs) with ART clinics have also shown improved health outcomes [19–21]; therefore, we believe that addition and improvement of service lines in ART clinics may be beneficial to case finding.

The UNAIDS “90–90-90” strategy still faces challenges. The HIV epidemic among MSM is a major public health concern in China [22], though community-based organizations have conducted HIV testing campaigns and outreach efforts through in-person and online social networking means [23]. Integration of partner testing with HIV services needs to be further strengthened in order to control the spread of HIV.

### Limitations

This study has some important limitations. First, the reported testing rate is likely an underestimate. Partners may have sought testing elsewhere or outside of the study period. Only partner testing that occurred through consultation with the index participant’s case manager was included in our analysis. Second, the lack of a control group does not allow us to directly compare the HIV testing rate in our pilot study to the standard of care. Third, to protect the privacy of the partners referred by index participants, very little information was collected about them (e.g. lacking information on average counseling duration of case managers and intervention fidelity). Finally, the standard case definition for an adult HIV infection diagnosis in China requires a positive result on a confirmatory western blot antibody test. However, we considered referred partners to be HIV-positive for the purposes of this study if they tested positive for HIV through ELISA screening (i.e. without a western blot test).

## Conclusions

Our findings indicatethat integration of MSM partner testing with ART services by leveraging health communication capabilities was feasible for increasing partner referral testing and HIV case finding among MSM. Nurses as case managers can effectively encourage index participants to refer their sexual partners for HIV testing.

## Abbreviations

AHRs: Adjusted hazard ratios
AORs: Adjusted odds ratios
ART: Antiretroviral therapy
CIs: 95% confidence intervals
MSM: Men who have sex with men
NCAIDS: National Center for AIDS/STD Control and Prevention
